# Lymphatic vascular invasion: Diagnostic variability and overall survival impact on patients undergoing surgical resection

**DOI:** 10.1016/j.xjon.2024.08.012

**Published:** 2024-08-31

**Authors:** John Varlotto, Rick Voland, Negar Rassaei, Dani Zander, Malcolm M. DeCamp, Jai Khatri, Yousef Shweihat, Kemnasom Nwanwene, Maria Tria Tirona, Thomas Wright, Toni Pacioles, Muhammad Jamil, Khuram Anwar, John Flickinger

**Affiliations:** a Department of Oncology, Edwards Comprehensive Cancer Center/Marshall University, Huntington, WVa; b Department of Ophthalmology, University of Wisconsin, Madison, Wis; c Centers for Disease Control and Prevention, Atlanta, Ga; d Department of Pathology and Laboratory Medicine, University of Cincinnati, Cincinnati, Ohio; e Division of Cardiothoracic Surgery, University of Wisconsin School of Medicine and Public Health, Madison, Wis; f Department of Internal Medicine, Marshall Health, Huntington, WVa; g Department of Radiation Oncology, University of Pittsburgh Medical Center, Pittsburgh, Pa

**Keywords:** lymphatic vascular invasion, diagnostic variance, lung cancer surgery, prognosis

## Abstract

**Objective:**

The diagnostic criteria of lymphatic vascular invasion have not been standardized. Our investigation assesses the factors associated with lymphatic vascular invasion positive tumors and the impact of lymphatic vascular invasion on overall survival for patients with non–small cell lung cancer undergoing (bi)lobectomy with an adequate node dissection.

**Methods:**

The National Cancer Database was queried from the years 2010 to 2015 to find surgical patients who underwent lobectomy with at least 10 lymph nodes examined (adequate node dissection) and with known lymphatic vascular invasion status. Paired *t* tests were used to distinguish differences between the patients with and without lymphatic vascular invasion in their specimen. Multivariable analysis was used to determine factors associated with overall survival. Propensity score matching adjusting for overall survival factors was used to determine the lymphatic vascular invasion's overall survival impact by grade, histology, p-T/N/overall stage, and tumor size.

**Results:**

Lymphatic vascular invasion status was reported in 91.6% and positive in 23.4% of 28,842 eligible patients. Academic medical centers, institutions with populations more than 1,000,000, and the mid-Atlantic region reported higher rates of lymphatic vascular invasion positive tumors as well as overall survival compared with other cancer centers. Lymphatic vascular invasion was independently associated with a significant decrement in overall survival as per multivariable analysis and propensity score matching. Propensity score matching demonstrated that lymphatic vascular invasion was associated with a significant decrement in overall survival for all histologies, tumor grades, tumor sizes, and stages, except for more advanced pathologic stages T3/III/N2 and larger tumors greater than 4 cm for which overall survival was trending worse with lymphatic vascular invasion positive.

**Conclusions:**

Lymphatic vascular invasion positive varies based on hospital location/type and population, but it was associated with a decrement in overall survival that was independent of pathologic T/N/overall stage, histology, and tumor grade. Lymphatic vascular invasion must be standardized and considered as a staging variable and should be considered as a sole determinant for prognosis, especially for those with earlier-stage and smaller tumors.

**Figure:**
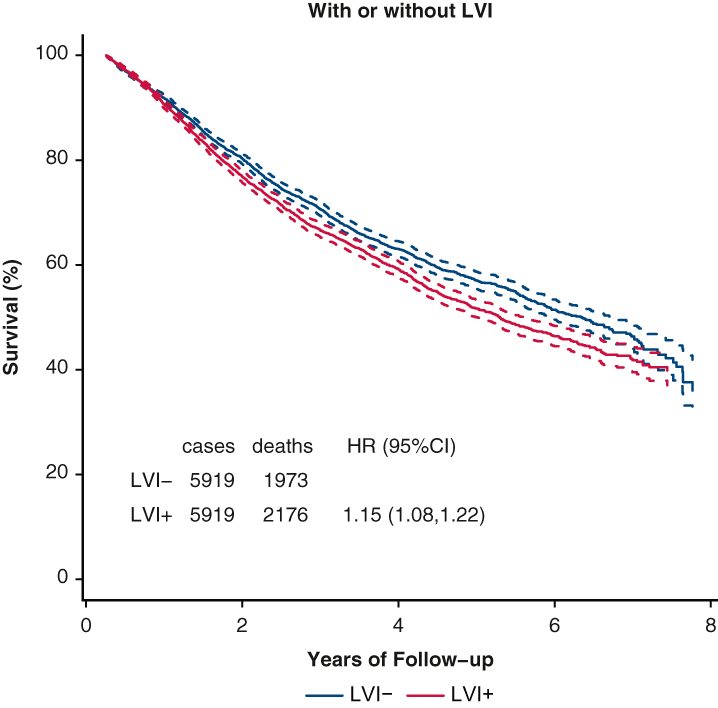
LVI and OS.

**Central Message**

LVI diagnosis varies by institution type, location, and surrounding population size. LVI adversely affects OS and significantly decreases survival in early-stage (I, II) disease as well as tumors less than 4 cm. Pathologic diagnosis of LVI needs to be standardized.

**Perspective**

We demonstrate that despite variance of the diagnosis of LVI throughout the United States, it remains an independent determinant of OS that is independent of tumor grade, histology, and early-stage tumors less than 4 cm.

Lymphatic vascular invasion (LVI) is a well-established negative prognostic factor for surgically resected non–small cell lung cancer (NSCLC)^1^, ^2^, ^3^ and has been associated with poor outcomes in locally advanced disease.^4^ Additionally, LVI has been correlated with lymph node involvement.^5^, ^6^, ^7^ The effects of LVI differ by series, with some investigations demonstrating a local recurrence (LR) risk,^1^^,^^5^, ^6^, ^7^ a distal recurrence (DR) risk,^2^^,^^4^^,^^8^ both LR/DR,^9^ and an overall survival (OS) decrement.^1^^,^^2^^,^^4^ A large meta-analysis of 87 surgical patient studies recently showed that LVI is associated with LR, DR, and OS.^10^ Despite its known association with the prognosis of patients with lung cancer, LVI has not been incorporated into lung cancer staging,^11^ but it is considered a risk factor for the consideration of adjuvant systemic therapy in the National Comprehensive Cancer Network guidelines based on consensus opinion of lower-level evidence (level 2A).^12^

The rates of pathologically reported LVI have varied greatly in the literature. Even in a stage I population, the rates of LVI can range from 5% to 49%.^3^ Therefore, we undertook this investigation to assess whether the rates of LVI positivity (LVI+) vary depending on the institution type, institution location, and patient population, as well as histologic factors. Furthermore, we wanted to assess whether the reported presence or absence of LVI affected the survival of surgical patients independently of tumor grade, N/T/overall stage, histology, and tumor size and whether it should be used as a sole criterion for the consideration of adjuvant therapy in patients undergoing an R0 resection with adequate node dissection.

In the recent past, the only established (neo)adjuvant therapy was chemotherapy, which was found to have a small 5-year survival benefit of 5.4%^13^ and was underused due to limited efficacy, toxicity, and prolonged surgical recovery.^13^ Recently, phase 3 trials of adjuvant and neoadjuvant approaches using immunotherapy have greatly improved outcomes and raised the benefit-to-risk ratio to allow for the consideration of (neo)adjuvant therapies in patients not traditionally considered for therapy.^14^, ^15^, ^16^, ^17^, ^18^ Unlike chemotherapy, adjuvant immunotherapy's effect on disease-free survival appeared to work better in lower-stage disease with pembrolizumab (hazard ratio [HR], 0.76 stage IB, HR, 0.70 stage II, HR, 0.92 stage IIIA).^15^ However, subset analysis noted that adjuvant atezolizumab demonstrated better PFS with higher stage (HR, 0.62 stage IIIA, HR, 0.77 stage IIB, HR, 0.73 stage IIA) and is approved in surgical patients with tumors having a PD-L1 greater than 1%.^14^ Interestingly, adjuvant pembrolizumab's effect on PFS was independent of PD-L1 and subset analysis noted the lowest HR in those having a PD-L1 of 1% to 49% versus those with a PD-L1 less than 1% and PD-L1 greater than 50%.^15^ Because of the recent improvements in adjuvant therapy, we think that investigating LVI's impact on survival may help clinicians to consider patients for adjuvant therapy who would not have traditional risk factors (tumor size ≥4 cm and node-positive disease) for consideration of adjuvant therapy. LVI+ as a selection tool in the neoadjuvant setting is difficult because it is rarely detected on biopsy specimens.

## Material and Methods

**Figure E1:**
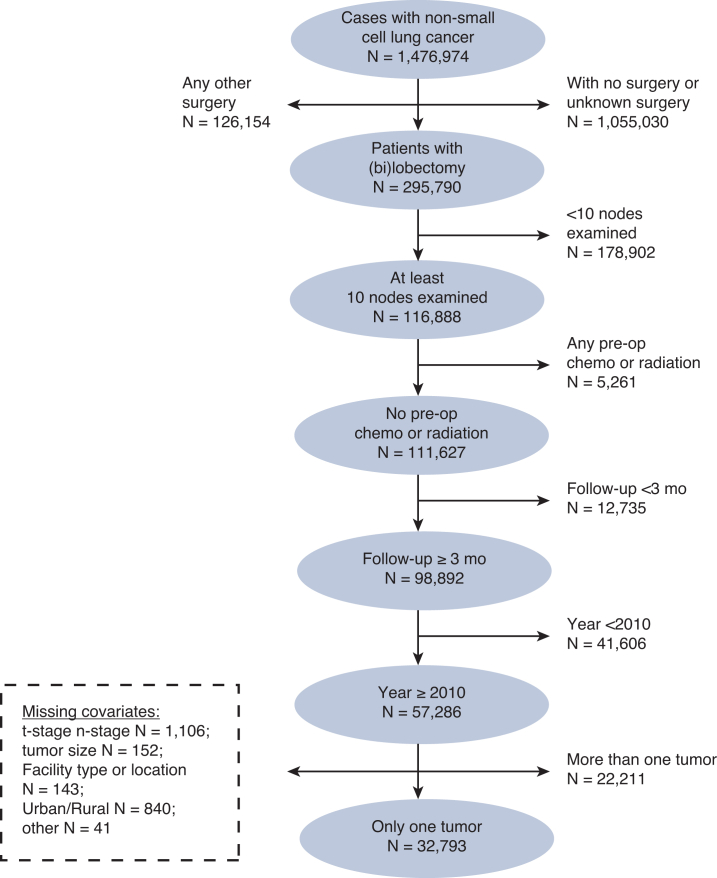
Patient selection criteria. *LVI*, Lymphatic vascular invasion.

Data included in this study were derived from the National Cancer Database (NCDB) registry for patients diagnosed between 2010 and 2015. The NCDB currently captures approximately 70% of all newly diagnosed malignancies in the United States. Because the patient information was deidentified, this investigation was exempted from Institutional Review Board approval. Individuals included were those undergoing (bi)lobectomy (LB) for stages I to III NSCLC. Exclusion criteria were any preoperative chemotherapy or radiation, follow-up less than 3 months, stage IV disease, and more than 1 tumor nodule. There were 25 patients with pN3 nodes, and they were also removed from the analyses. After all exclusions (Figure E1), 28,842 surgical patients were eligible for inclusion in our analysis. Although there are varying definitions of adequacy of pathologic node staging during resection of NSCLC, we chose the definition of those with examination of a minimum of 10 lymph nodes irrespective of node station location as per the American College of Surgeons Commission on Cancer NSCLC quality of surveillance criteria because this is the source of our data.^19^

The NCDB demarcated LVI beginning in 2010 as lymphatic invasion positive, lymphatic invasion negative, and unknown lymphatic invasion status. Patients with unknown LVI status were eliminated from our investigation. For the purpose of this study, LVI referred to the presence of tumor in endothelial-lined spaces, encompassing both lymphatic and vascular spaces. Multivariable analysis (MVA) was conducted to investigate factors potentially associated with OS. Factors analyzed included the following: age, sex, race (non-Hispanic White, White Hispanic, Black, Asian/Pacific Islander, other/unknown), tumor location (right upper lobe, right middle lobe, right lower lobe, left upper lobe, left lower lobe, other, not otherwise specified), histology (adenocarcinoma, bronchioloalveolar, adenosquamous cell carcinoma, large cell carcinoma, squamous cell carcinoma), tumor grade (well, moderate, poor, undifferentiated/anaplastic), tumor size, number of nodes examined, number of positive lymph nodes, facility type (community program, academic/research institute, comprehensive community cancer program, integrated cancer network), facility location (New England, East North Central, East South Central, Middle Atlantic, Mountain, Pacific, South Atlantic, West North Central, West South Central), patient's income (<$38,000; $38,000-$47,999; >48,000-$62,999; >$63,000), patient's insurance (unknown, commercial, Medicaid, Medicare, no insurance, other government), high school graduation rate (counties with ≥7% or <7% without a diploma), population size (≥1 million population, <1 million), year of diagnosis (2010, 2011, 2012, 2013, 2014, 2015), treatment (bi)lobectomy or bilobectomy with chest wall resection, LB with diaphragm resection, LB with pericardial resection, LB extended, not otherwise specified, LB with mediastinal lymph node dissection, LB, pathologic(p)N stage (N0-3), pathologic(p)T stage (T1-4), use of radiation, use of chemotherapy, and surgical margin status (positive, unknown, and negative).

### Statistical Analyses

The data were assessed for those surgical specimens with a diagnosis of LVI or no LVI in the total population. A paired *t* test was performed to assess the differences between those populations with or without a known diagnosis of LVI.^20^

To decrease the risk of a spurious positive event in our multivariate analysis, analysis of variance (ANOVA) tests^21^ were performed for factors associated with OS. Only when factors were positive in both the ANOVA and MVA were they considered positive. Cox regression analysis was used for the OS MVA. HRs were generated for OS.

Propensity score matching (PSM), adjusting for factors associated with OS, was used to determine LVI's impact by tumor size, grade, histology, overall pathologic stage, pT stage, and pNode stage (N-stage). All-cause survival was the primary outcome. Analyses were conducted using R-4.3.2. Factors used in matching were determined by examining effects from MVA logistic regression for LVI positive versus LVI negative and effects from MVA Cox analysis of OS with LVI positive versus LVI negative. LVI-positive and LVI-negative cases were matched 1:1 without replacement using nearest-neighbor matching in the MatchIT package with a maximum distance (caliper) of 0.20, matching by closest distance first, and using distances calculated in the Twang package.^22^ Exact matches were forced for subgroup analyses. Covariate balance was assessed using Love plots to determine that standardized mean differences were less than 0.10 after matching both for individual factors and for overall distance.

Kaplan–Meier plots^23^ of OS were used for the matched data to show the final analysis instead of multivariate Cox regression (for the final analyses) to obtain marginal HR estimates instead of Cox regression of the whole sample. CIs for HRs were determined using robust standard errors in Cox regression on the matched pairs because this method provides marginal HR estimates and does not assume independence within the matched pairs. Analyzing matched pairs differs from MVA Cox regression of the whole sample.^24^^,^^25^

## Results

A total of 28,842 patients undergoing LB were eligible for our study with a median follow-up of (41.3 months). During the follow-up period, 8014 deaths occurred. LVI status was determined in 91.6% of surgical specimens. Of those specimens for which LVI was reported, 6777 patients (23.4%) exhibited LVI in their surgical specimens and 22,065 patients (76.6%) did not. LVI was found in 48.9% of specimens with positive nodes, and 14.2% of specimens with negative nodes had LVI. A complete list of demographic factors and their comparison can be found in Table 1 for those with LVI-negative and LVI-positive tumors. LVI positivity was associated with a younger age, male, non-Asian race, adenosquamous/nonbronchioloalveolar histology/large cell neuroendocrine carcinoma/nonsquamous histologies compared with adenocarcinoma, differentiations other than well, number of nodes pathologically positive, academic setting, Mid-Atlantic institution location, larger patient population areas (>1,000,000), pT2 or higher T-stage, pN1/N2 stage, positive surgical margins, and pleural/rib/brachial plexus/pericardial extension.

**Table 1:** Demographic of patient groups with and without lymphatic vascular invasion

Variables	Overall	LVI–	LVI+	OR	Lower 95% for OR	Upper 95% for OR	*P* value
	(N = 28,842)	(N = 22,065)	(N = 6777)				
Age at diagnosis (y)							
Mean (SD)	**66.8 (9.34)**	**66.8 (9.34)**	**66.6 (9.36)**	**1.006**	**1.003**	**1.010**	**<.001**
Median [Min, Max]	67.0 [40.0, 90.0]	67.0 [40.0, 90.0]	67.0 [40.0, 90.0]	—			
Sex							
Male	13,827 (47.9%)	10,366 (47.0%)	3461 (51.1%)	(ref)			
Female	**15,015 (52.1%)**	**11,699 (53.0%)**	**3316 (48.9%)**	**0.904**	**0.849**	**0.961**	**.001**
Race_Ethnicity							
White Non-Hispanic	23,814 (82.6%)	18,244 (82.7%)	5570 (82.2%)				
Hispanic	683 (2.4%)	510 (2.3%)	173 (2.6%)	0.970	0.792	1.184	.768
Black	2286 (7.9%)	1728 (7.8%)	558 (8.2%)	0.982	0.876	1.100	.758
Asian	**858 (3.0%)**	**661 (3.0%)**	**197 (2.9%)**	**0.786**	**0.650**	**0.947**	**.012**
Other	1201 (4.2%)	922 (4.2%)	279 (4.1%)	0.942	0.807	1.097	.449
CDCCs							
0	14,687 (50.9%)	11,198 (50.8%)	3489 (51.5%)	(ref)			
1	9750 (33.8%)	7467 (33.8%)	2283 (33.7%)	1.036	0.968	1.109	.306
2	3314 (11.5%)	2574 (11.7%)	740 (10.9%)	0.978	0.883	1.081	.660
≥3	1091 (3.8%)	826 (3.7%)	265 (3.9%)	1.113	0.946	1.305	.192
Histology							
Adenocarcinoma	16,549 (57.4%)	12,577 (57.0%)	3972 (58.6%)	(ref)			
Adenosquamous	**720 (2.5%)**	**486 (2.2%)**	**234 (3.5%)**	**1.253**	**1.046**	**1.497**	**.014**
Bronchoalveolar	**1569 (5.4%)**	**1404 (6.4%)**	**165 (2.4%)**	**0.674**	**0.559**	**0.807**	**<.001**
Large cell	**758 (2.6%)**	**574 (2.6%)**	**184 (2.7%)**	**0.833**	**0.685**	**1.008**	**.063**
Large cell neuroendocrine	**359 (1.2%)**	**234 (1.1%)**	**125 (1.8%)**	**1.558**	**1.216**	**1.988**	**<.001**
Pulmonary sarcomatoid	255 (0.9%)	172 (0.8%)	83 (1.2%)	1.050	0.778	1.406	.749
Salivary gland	49 (0.2%)	41 (0.2%)	8 (0.1%)	0.553	0.213	1.276	.191
Squamous	**8583 (29.8%)**	**6577 (29.8%)**	**2006 (29.6%)**	**0.902**	**0.839**	**0.970**	**.005**
Grade differentiation							
Well	**3704 (12.8%)**	**3457 (15.7%)**	**247 (3.6%)**	**(ref)**			
Moderate	**12,935 (44.8%)**	**10,124 (45.9%)**	**2811 (41.5%)**	**2.550**	**2.210**	**2.953**	**<.001**
Poor	**10,253 (35.5%)**	**7099 (32.2%)**	**3154 (46.5%)**	**3.430**	**2.965**	**3.983**	**<.001**
Undifferentiated	**345 (1.2%)**	**242 (1.1%)**	**103 (1.5%)**	**3.457**	**2.550**	**4.667**	**<.001**
Not determined	**1605 (5.6%)**	**1143 (5.2%)**	**462 (6.8%)**	**3.115**	**2.593**	**3.748**	**<.001**
Tumor_size (mm)							
Mean (SD)	34.6 (27.9)	33.2 (27.2)	38.9 (29.9)	1.000	0.998	1.001	.579
Median [Min, Max]	28.0 [1.00, 950]	27.0 [1.00, 950]	33.0 [1.00, 950]	—			
Nodes positive							
Mean (SD)	**0.876 (2.25)**	**0.488 (1.56)**	**2.14 (3.39)**	**1.120**	**1.100**	**1.140**	**<.001**
Median [Min, Max]	0 [0, 61.0]	0 [0, 50.0]	1.00 [0, 61.0]	—			
Facility_type							
Academic Program	11,894 (41.2%)	8782 (39.8%)	3112 (45.9%)	(ref)			
Community Cancer Program	**1612 (5.6%)**	**1250 (5.7%)**	**362 (5.3%)**	**0.770**	**0.668**	**0.885**	**<.001**
Comprehensive Cancer Center	**10,978 (38.1%)**	**8644 (39.2%)**	**2334 (34.4%)**	**0.768**	**0.714**	**0.826**	**<.001**
Integrated Network Cancer Program	**4358 (15.1%)**	**3389 (15.4%)**	**969 (14.3%)**	**0.804**	**0.732**	**0.883**	**<.001**
Facility_location							
Mid Atlantic	5308 (18.4%)	3663 (16.6%)	1645 (24.3%)	(ref)			
East North Central	**5087 (17.6%)**	**3995 (18.1%)**	**1092 (16.1%)**	**0.622**	**0.562**	**0.689**	**<.001**
East South Central	**2447 (8.5%)**	**1937 (8.8%)**	**510 (7.5%)**	**0.604**	**0.529**	**0.688**	**<.001**
Mountain	**985 (3.4%)**	**797 (3.6%)**	**188 (2.8%)**	**0.610**	**0.502**	**0.738**	**<.001**
New England	**1592 (5.5%)**	**1220 (5.5%)**	**372 (5.5%)**	**0.680**	**0.587**	**0.787**	**<.001**
Pacific	**2786 (9.7%)**	**2155 (9.8%)**	**631 (9.3%)**	**0.621**	**0.549**	**0.702**	**<.001**
South Atlantic	**6670 (23.1%)**	**5269 (23.9%)**	**1401 (20.7%)**	**0.600**	**0.544**	**0.660**	**<.001**
West North Central	**2137 (7.4%)**	**1677 (7.6%)**	**460 (6.8%)**	**0.572**	**0.500**	**0.654**	**<.001**
West South Central	**1830 (6.3%)**	**1352 (6.1%)**	**478 (7.1%)**	**0.809**	**0.705**	**0.927**	**.002**
Urban/rural population							
>1 million	15,111 (52.4%)	11,312 (51.3%)	3799 (56.1%)	(ref)			
<1 million	**13,731 (47.6%)**	**10,753 (48.7%)**	**2978 (43.9%)**	**0.849**	**0.796**	**0.906**	**<.001**
Year of diagnosis							
2010	3781 (13.1%)	2879 (13.0%)	902 (13.3%)	(ref)			
2011	4106 (14.2%)	3112 (14.1%)	994 (14.7%)	1.064	0.948	1.195	.291
2012	4324 (15.0%)	3305 (15.0%)	1019 (15.0%)	0.972	0.866	1.090	.627
2013	4720 (16.4%)	3607 (16.3%)	1113 (16.4%)	1.032	0.922	1.155	.584
2014	5383 (18.7%)	4116 (18.7%)	1267 (18.7%)	1.013	0.907	1.130	.823
2015	6528 (22.6%)	5046 (22.9%)	1482 (21.9%)	0.991	0.891	1.102	.865
pT_stage							
T0	18 (0.1%)	16 (0.1%)	2 (0.0%)	0.166	0.026	0.599	.018
T1	12,912 (44.8%)	10,882 (49.3%)	2030 (30.0%)	(ref)			
T2	**12,882 (44.7%)**	**9128 (41.4%)**	**3754 (55.4%)**	**1.381**	**1.274**	**1.497**	**<.001**
T3	**2820 (9.8%)**	**1929 (8.7%)**	**891 (13.1%)**	**1.426**	**1.223**	**1.663**	**<.001**
T4	**210 (0.7%)**	**110 (0.5%)**	**100 (1.5%)**	**1.975**	**1.298**	**3.000**	**.001**
n_stage_path							
N0	21,112 (73.2%)	18,113 (82.1%)	2999 (44.3%)	(ref)			
N1	**4710 (16.3%)**	**2582 (11.7%)**	**2128 (31.4%)**	**3.193**	**2.915**	**3.497**	**<.001**
N2	**3020 (10.5%)**	**1370 (6.2%)**	**1650 (24.3%)**	**3.556**	**3.141**	**4.024**	**<.001**
Surgical margins							
Margin negative	27,565 (95.6%)	21,336 (96.7%)	6229 (91.9%)	(ref)			
Margin positive	**1146 (4.0%)**	**645 (2.9%)**	**501 (7.4%)**	**1.489**	**1.291**	**1.716**	**<.001**
Unknown margins	131 (0.5%)	84 (0.4%)	47 (0.7%)	1.382	0.913	2.067	.120
Site of surgery							
Lobectomy with mediastinal node dissection	24,991 (86.6%)	19,206 (87.0%)	5785 (85.4%)	(ref)			
(Bi)lobectomy extended, NOS	798 (2.8%)	560 (2.5%)	238 (3.5%)	1.083	0.909	1.287	.368
(Bi)lobectomy with chest wall	474 (1.6%)	336 (1.5%)	138 (2.0%)	0.965	0.751	1.235	.778
(Bi)lobectomy with diaphragm	26 (0.1%)	14 (0.1%)	12 (0.2%)	1.349	0.572	3.139	.487
(Bi)lobectomy with pericardium	52 (0.2%)	31 (0.1%)	21 (0.3%)	1.294	0.686	2.400	.418
(Bi)lobectomy	2501 (8.7%)	1918 (8.7%)	583 (8.6%)	1.049	0.939	1.169	.396
Extension							
1 lung	20,888 (72.4%)	16,906 (76.6%)	3982 (58.8%)	(ref)			
Bronchus	535 (1.9%)	374 (1.7%)	161 (2.4%)	0.974	0.788	1.199	.804
Entire lung	29 (0.1%)	20 (0.1%)	9 (0.1%)	1.156	0.474	2.641	.738
Hilar region	294 (1.0%)	210 (1.0%)	84 (1.2%)	0.976	0.733	1.291	.867
Great vessels/aorta	223 (0.8%)	120 (0.5%)	103 (1.5%)	1.336	0.893	1.992	.156
Pleura	**5646 (19.6%)**	**3616 (16.4%)**	**2030 (30.0%)**	**1.755**	**1.618**	**1.904**	**<.001**
Ribs	**123 (0.4%)**	**79 (0.4%)**	**44 (0.6%)**	**1.741**	**1.117**	**2.684**	**.013**
Brachial plexus	**1043 (3.6%)**	**710 (3.2%)**	**333 (4.9%)**	**1.316**	**1.083**	**1.598**	**.006**
Heart/visceral pericardium	**61 (0.2%)**	**30 (0.1%)**	**31 (0.5%)**	**2.065**	**1.151**	**3.709**	**.015**
Any lung radiation							
No	25,826 (89.5%)	20,353 (92.2%)	5473 (80.8%)	(ref)			
Yes	3016 (10.5%)	1712 (7.8%)	1304 (19.2%)	0.996	0.897	1.105	.935
Any lung chemotherapy							
No	19,134 (66.3%)	16,114 (73.0%)	3020 (44.6%)	(ref)			
Yes	**9708 (33.7%)**	**5951 (27.0%)**	**3757 (55.4%)**	**1.146**	**1.057**	**1.242**	**.001**

Statistical significance (< .05) between those diagnosed with and without LVI. *LVI*, Lymphatic vascular invasion; *OR*, odds ratio; *CDCC*, Charlston-Deyo Score; *NOS*, not otherwise specified.

The ANOVA (Table E1) and MVA tests (Table 2) demonstrated factors associated with OS in our population. By multivariable modeling, a lower chance of OS was noted with older age, men, non-Hispanic/non-Asian race, increasing Charlson comorbidity (Charlston-Deyo Score), adenosquamous/large cell/large cell neuroendocrine/sarcomatoid/squamous cell histologies compared with adenocarcinomas, tumor differentiation other than well, pathologic tumor size, number of positive nodes, nonacademic medical centers, non-Mid Atlantic medical centers, areas with smaller populations (<1,000,000 people), diagnosis in years other than 2015, pT2-4 stage, pN1-2 stage, positive surgical margins, use of radiation, not receiving adjuvant chemotherapy, and LVI+ tumors. Because all factors associated with OS were seen in the ANOVA, all were considered to be significant. Please note that type of surgery, LB with chest-wall resection, was positive in the MVA, but was noted to be nonsignificant by the ANOVA; therefore, this finding was found to be spurious.

**Table 2:** Multivariate analysis for overall survival

Variables	HR	2.5%	97.5%	*P*
Age at diagnosis (y)	1.030	1.028	1.033	<.001
Sex: female	0.745	0.712	0.779	<.001
Sex: male (ref)				
Race: White Non-Hispanic(ref)				
Race: Hispanic	**0.829**	**0.705**	**0.977**	**.025**
Race: Black	0.983	0.9	1.072	.692
Race: Asian	**0.744**	**0.633**	**0.874**	**<.001**
Race: Other	0.949	0.851	1.058	.346
CDCC: 0 (ref)				
CDCC: 1	**1.140**	**1.084**	**1.198**	**<.001**
CDCC: 2	**1.392**	**1.301**	**1.489**	**<.001**
CDCC: ≥3	**1.568**	**1.413**	**1.739**	**<.001**
Histology: Adenocarcinoma (ref)				
Histology: Adenosquamous	**1.258**	**1.109**	**1.428**	**<.001**
Histology: Bronchoalveolar	1.049	0.937	1.175	.404
Histology: Large cell	**1.342**	**1.18**	**1.526**	**<.001**
Histology: Large cell neuroendocrine	**1.950**	**1.648**	**2.309**	**<.001**
Histology: Pulmonary sarcomatoid	**1.670**	**1.376**	**2.027**	**<.001**
Histology: Salivary gland	0.483	0.216	1.079	.076
Histology: Squamous	**1.159**	**1.101**	**1.22**	**<.001**
Grade: Well differentiated (ref)				
Grade: Moderately differentiated	**1.323**	**1.207**	**1.451**	**<.001**
Grade: Poorly differentiated	**1.416**	**1.287**	**1.557**	**<.001**
Grade: Undifferentiated	**1.381**	**1.126**	**1.693**	**.002**
Grade: Not determined	**1.327**	**1.163**	**1.514**	**<.001**
Tumor size (per 1 mm)	**1.002**	**1.001**	**1.003**	**<.001**
Positive nodes	**1.045**	**1.037**	**1.053**	**<.001**
Facility: Academic (ref)				
Facility: Community Cancer Program	**1.266**	**1.151**	**1.392**	**<.001**
Facility: Comprehensive Community Center	**1.103**	**1.047**	**1.163**	**<.001**
Facility: Integrated Network Cancer Program	**1.065**	**0.995**	**1.141**	**.071**
Facility location: Mid Atlantic (ref)				
Facility location: East North Central	**1.239**	**1.147**	**1.338**	**<.001**
Facility location: East South Central	**1.272**	**1.159**	**1.397**	**<.001**
Facility location: Mountain	1.104	0.962	1.267	.158
Facility location: New England	1.057	0.946	1.182	.327
Facility location: Pacific	**1.174**	**1.069**	**1.288**	**.001**
Facility location: South Atlantic	**1.198**	**1.112**	**1.29**	**<.001**
Facility location: West North Central	**1.208**	**1.096**	**1.331**	**<.001**
Facility location: West South Central	1.052	0.943	1.172	.365
Urban/rural >1 million local population (ref)				
Urban/rural <1 million local population	**1.073**	**1.025**	**1.124**	**.003**
Diagnosis year 2010 (ref)				
Diagnosis year 2011	1.031	0.961	1.105	.395
Diagnosis year 2012	0.935	0.869	1.006	.073
Diagnosis year 2013	0.989	0.917	1.068	.784
Diagnosis year 2014	0.932	0.86	1.01	.087
Diagnosis year 2015	**0.884**	**0.81**	**0.966**	**.006**
pT-stage T0	0.877	0.363	2.117	.770
pT-stage-path T1 (ref)				
pT-stage-path T2	**1.276**	**1.205**	**1.35**	**<.001**
pT-stage-path T3	**1.746**	**1.59**	**1.917**	**<.001**
pT-stage-path T4	**1.788**	**1.332**	**2.401**	**<.001**
pN-stage-path N0 (ref)				
pN-stage-path N1	**1.544**	**1.455**	**1.639**	**<.001**
pN-stage-path N2	**1.957**	**1.817**	**2.107**	**<.001**
Surgical margins negative (ref)				
Surgical margins +	**1.465**	**1.339**	**1.603**	**<.001**
Surgical margins unknown	1.130	0.861	1.483	.379
Surgical resection: (bi)lobectomy with mediastinal node resection (ref)				
Surgical resection: (Bi)lobectomy extended NOS	1.102	0.978	1.241	.112
Surgical resection: (Bi)lobectomy with chest wall resection	**1.227**	**1.047**	**1.438**	**.012**
Surgical resection: (Bi)lobectomy with diaphragm resection	1.155	0.68	1.96	.594
Surgical resection: (Bi)lobectomy with pericardium resection	1.439	0.944	2.192	.091
Surgical resection: (Bi)lobectomy	0.996	0.923	1.074	.909
Extension: 1 lung (ref)				
Extension: bronchus	0.901	0.775	1.047	.174
Extension: entire lung	0.723	0.408	1.281	.267
Extension: hilar region	1.008	0.827	1.23	.933
Extension: great vessels/aorta	1.025	0.772	1.36	.866
Extension: pleura	**1.110**	**1.023**	**1.203**	**.012**
Extension: ribs	1.106	0.83	1.473	.493
Extension: brachial plexus	**1.170**	**1.034**	**1.323**	**.013**
Extension: heart/visceral pericardium	0.733	0.47	1.144	.171
LVI– (ref)				
LVI+	**1.191**	**1.13**	**1.255**	**<.001**
Lung chemotherapy: Yes (vs ref)	0.690	0.651	0.731	<.001
Lung chemotherapy: (ref = No)	1.000			
Lung radiation: Yes (vs ref)	1.228	1.143	1.319	<.001
Lung radiation: (ref = No)	1.000			

Statistical significance (< .05) between those diagnosed with and without LVI. *CDCC*, Charlston-Deyo Score; *HR*, hazard ratio; *LVI*, lymphatic vascular invasion; *NOS*, not otherwise specified.

**Figure 1:**
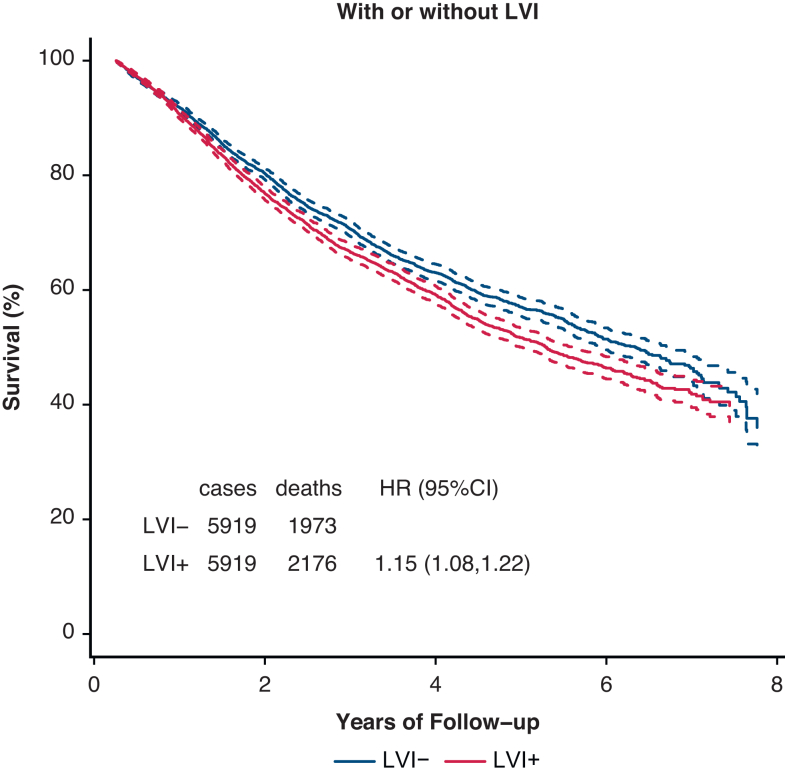
OS versus time (months) by LVI status. *LVI*, Lymphatic vascular invasion; *HR*, hazard ratio.

**Figure 2:**
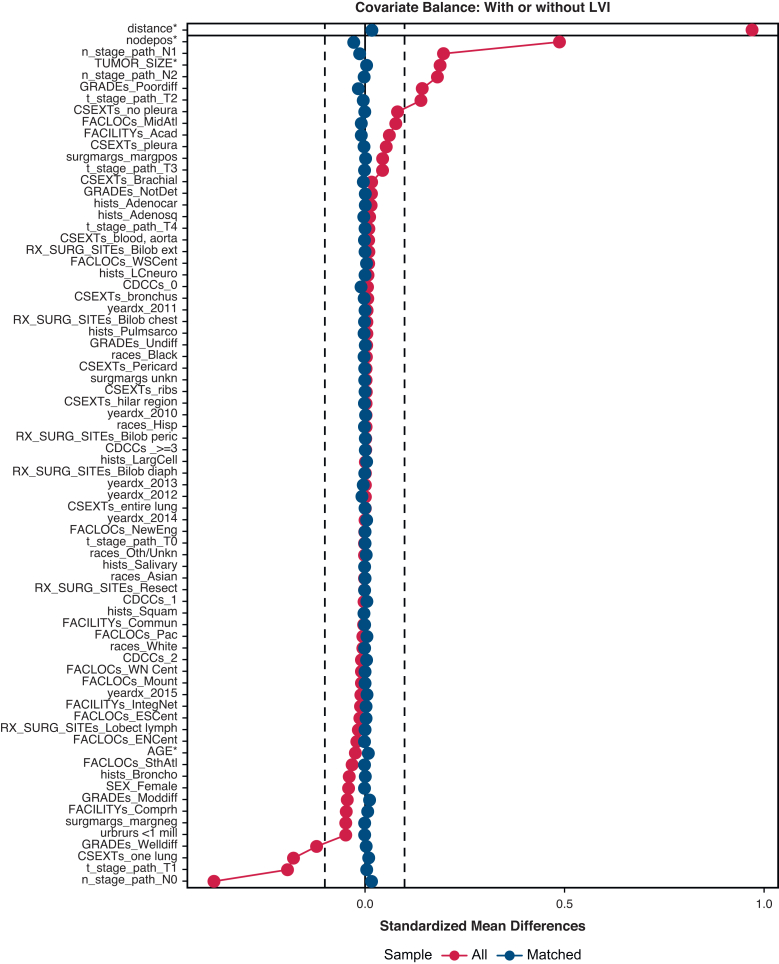
Dove plot associated with LVI status versus OS propensity match. *LVI*, Lymphatic vascular invasion.

**Figure 3:**
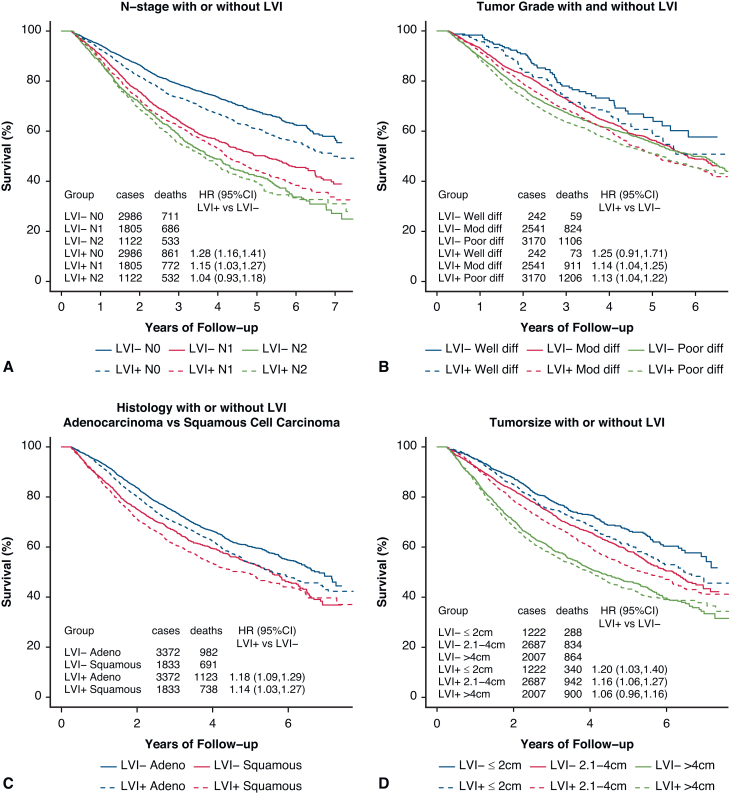

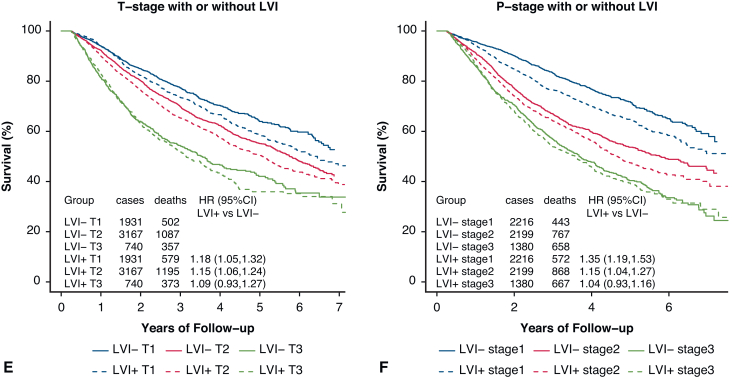
A, Propensity score–adjusted OS by nodal stage. B, Propensity score–adjusted OS by tumor grade. C, Propensity score–adjusted OS by histology. D, Propensity score–adjusted OS by tumor size. E, Propensity score–adjusted OS by pTStage. F, Propensity score–adjusted OS by pathologic overall stage. *LVI*, Lymphatic vascular invasion; *HR*, hazard ratio.

**Figure E2:**
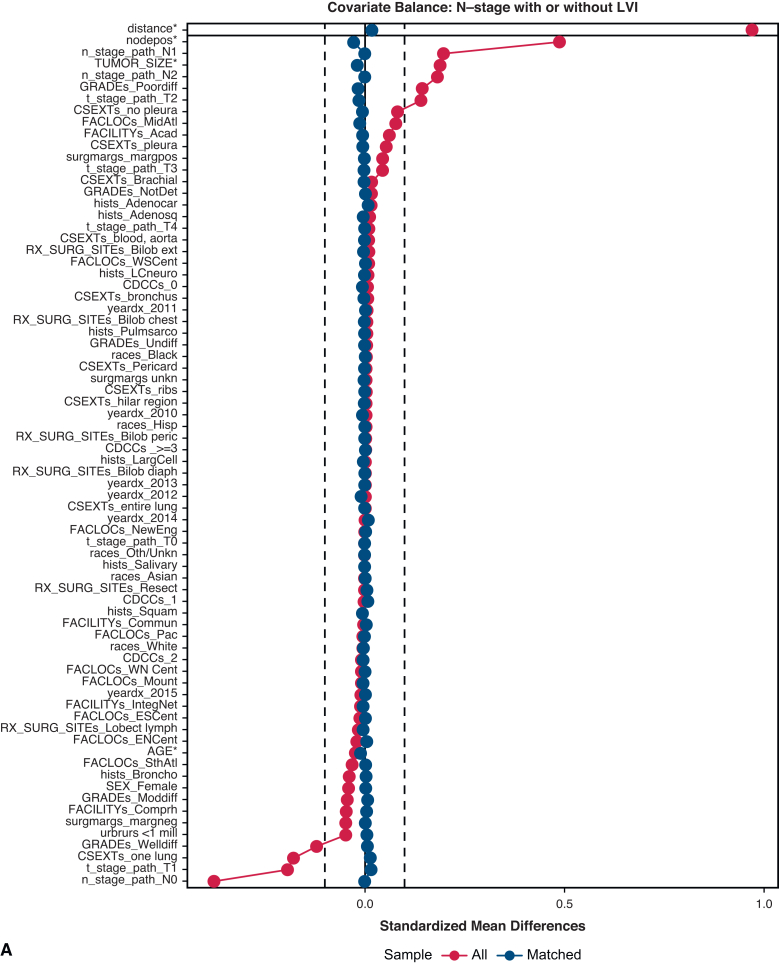

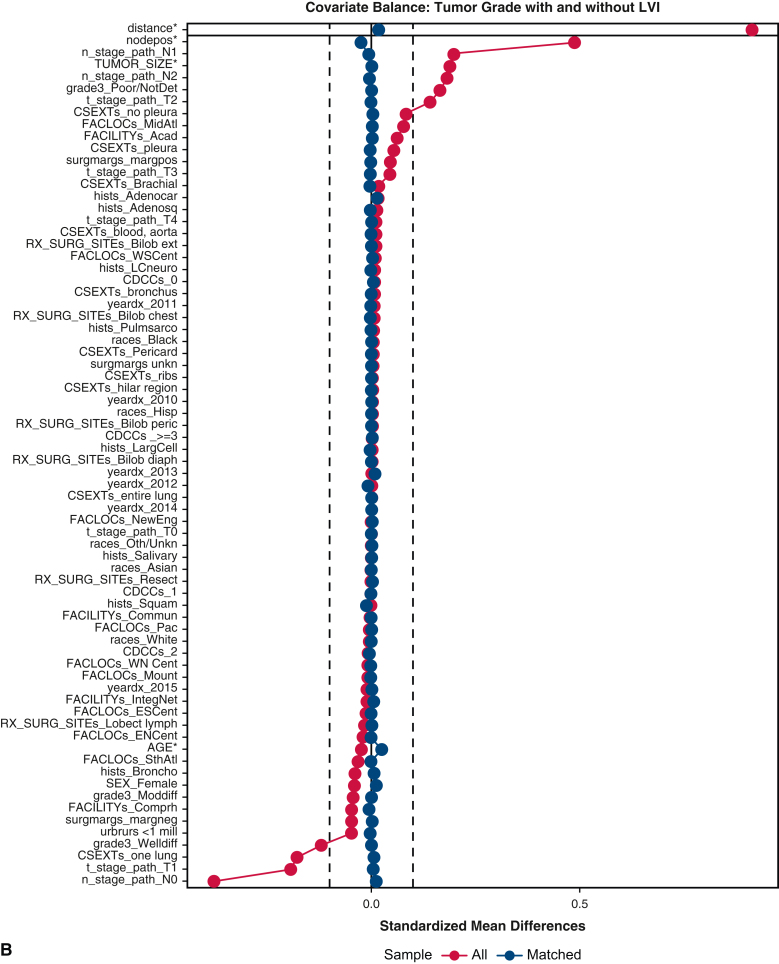

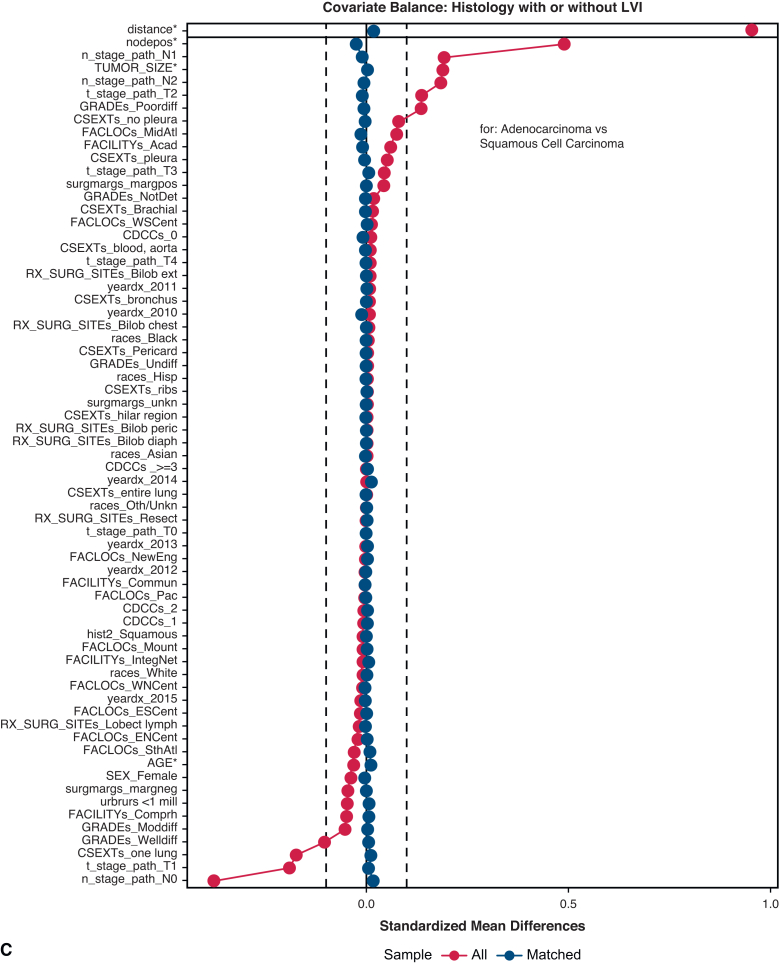

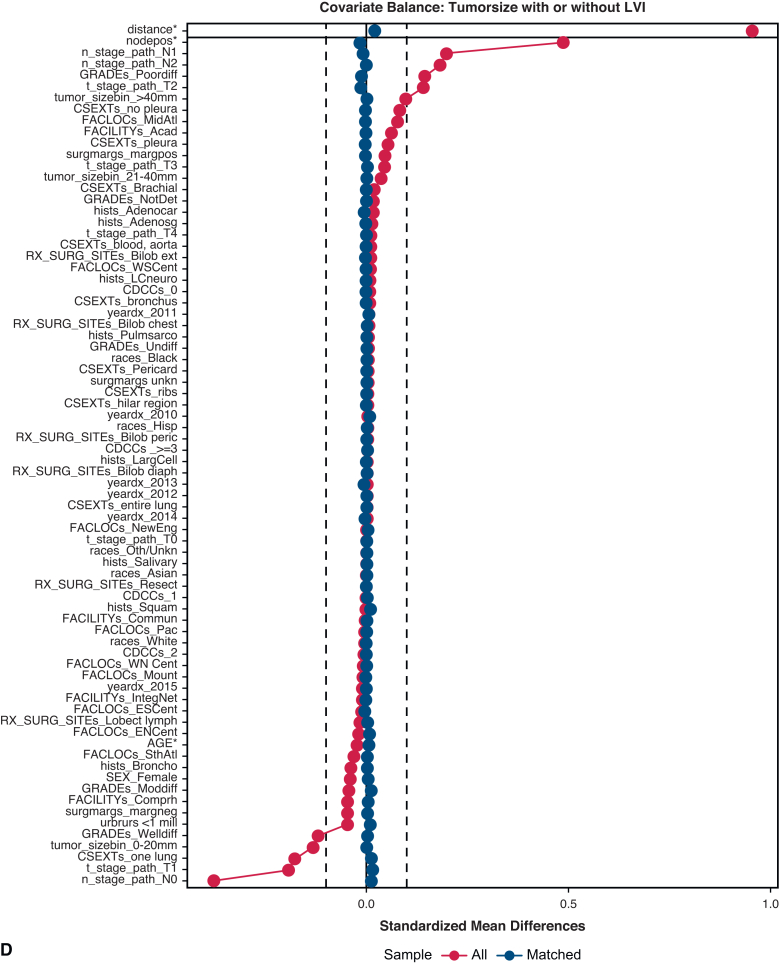

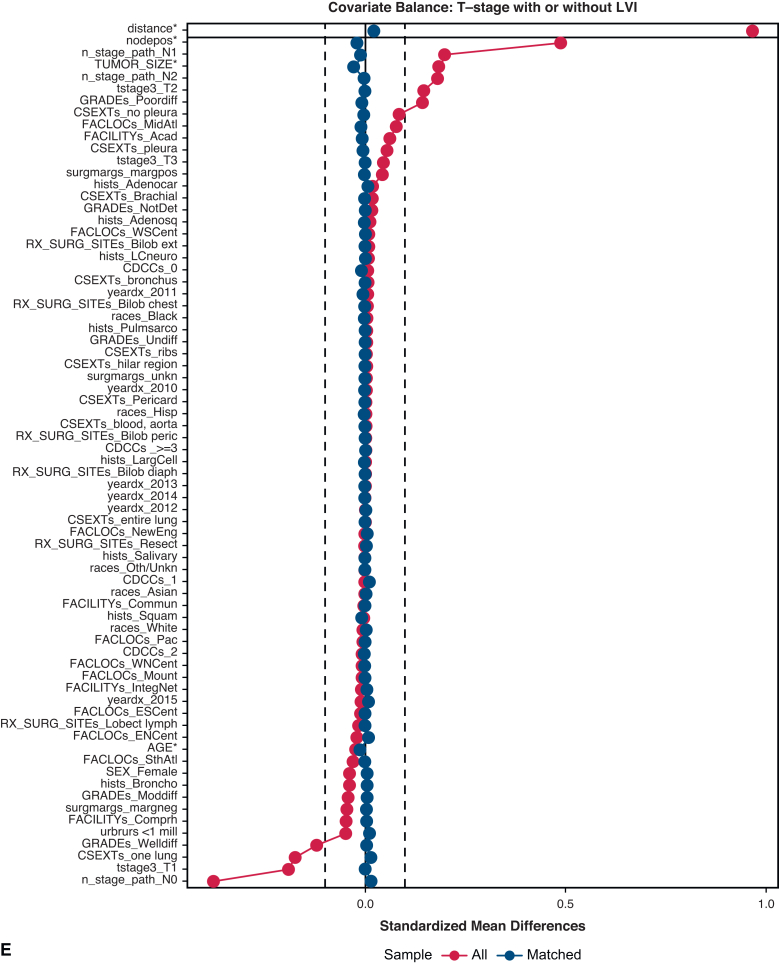

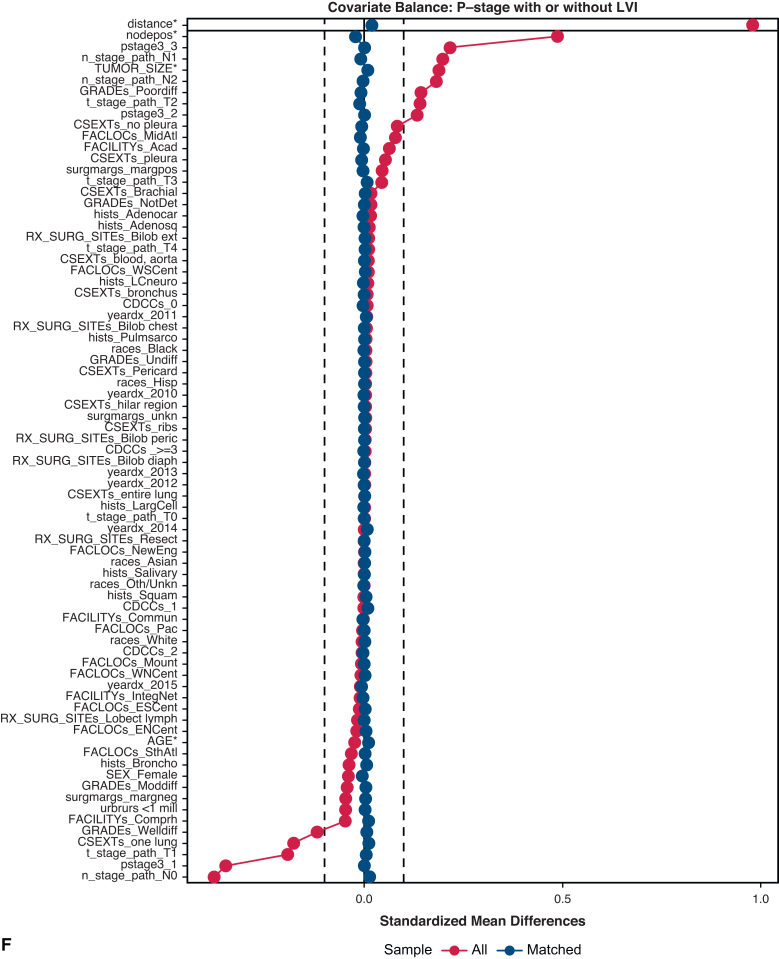
A, Love plot showing matching of characteristics used for propensity match for pN stage. B, Love plot showing matching of characteristics used for propensity match for tumor grade. C, Love plot showing matching of characteristics used for propensity match for histology. D, Love plot showing matching of characteristics used for propensity match for tumor size. E, Love plot showing matching of characteristics used for propensity match for pT stage. F, Love plot showing matching of characteristics used for propensity match for p stage. *LVI*, Lymphatic vascular invasion.

PSM by characteristics affecting OS was used to assess the impact of LVI on OS in Figure 1. The associated Love plot for this LVI curve is shown in Figure 2. The Love plots for LVI and its effect on OS in Figure 2 demonstrate that standardized mean differences were less than 0.10 after matching both for individual factors and for overall distance, and this shows the robustness of the matching process. LVI was significantly associated with an OS decrement (HR, 1.15; 1.08-1.22). PSM curves for OS were also generated for pTstage, tumor size, pNstage, pStage, grade, and histology are shown in Figure 3, *A-F* (the associated Love plots are shown in Figure E2, *A-F*). Tumor specimens with LVI adversely affected OS for both squamous cell and adenocarcinoma histologies as well as all differentiations. For N-stage, tumor size, T-stage, and overall stage, LVI had a significant effect on OS for the earlier stages/smaller sizes, but lost significance with higher stages/larger sizes as follows, listed by variable and HR with significance demarcated by an ∗: N0 - 1.28∗/N1 - 1.15∗/N2 - 1.04; stage 1 - 1.35∗/stage 2 - 1.15∗/stage 3 - 1.04; T1 - 1.18∗/T2∗ - 1.15/T3 - 1.09; and ≤2 cm - 1.20∗/2.1-4 cm - 1.16∗/>4 cm 1.06. Table E2 displays these variables and their HRs (95% CI).

## Discussion

This study focused on analyzing the relationship between LVI and prognosis. It should be noted that our study uses LVI as a single diagnostic category and does not separate lymphatic and vascular invasion as in other studies.^26^^,^^27^ LVI is usually defined as tumor cells within the lumen of a lymphatic channel as detected during histologic evaluation with standard hematoxylin and eosin staining.^28^ Although endothelium-lined lymphatic and vascular channels can in some cases be differentiated from each other, in practice distinction between these types of channels can be quite difficult.^27^ Immunostaining with lymphatic endothelium-specific marker D2-40^29^ and the panendothelial marker CD34^30^ has been used to distinguish between lymphatic and vascular invasion, respectively, but these stains are not routinely performed. Likewise, elastic stains have been used in some institutions to distinguish lymphatic from vascular structures. In theory, the elastic fibers should not be present in lymphatic vessels, but this staining method is more reliable for detecting tumor cells in vessels than its ability to differentiate type of vessel invasion.^31^ As per past investigations,^5^, ^6^, ^7^ LVI+ was more likely to be found in surgical specimens with positive lymph nodes than those with negative nodes.

It is no surprise that LVI was positive in specimens with poor histologic characteristics. LVI was associated with tumor grades other than well-differentiated, adenosquamous/squamous cell carcinomas/large cell neuroendocrine histologies, number of nodes positive, pTStages other than T1, N1/N2 disease rather than N0, positive surgical margins, and invasive tumors (invading pleura, ribs, brachial plexus, and visceral pericardium/heart). As expected, LVI was less like to be found in bronchoalveolar tumors, but it is puzzling to find LVI to be significantly less likely to be in large cell carcinoma specimens than adenocarcinoma. It is interesting to note that female and Asian patients are less likely to have LVI in their surgically resected specimens, and these populations have been found to have good prognostic characteristics such as a greater likelihood of nonsmoking-related cancers^32^ and a lower incidence of loco-regional failure, respectively.^10^

It is of great interest that LVI was more likely to be positive in the Mid-Atlantic area than all other 8 areas within the United States. We do not know of any other investigation that shows this regional phenomenon. Presently, it is unknown why the Mid-Atlantic area has a higher rate of LVI+. However, we speculate that this difference could be related to cultural variations influencing practice patterns within pathologist and oncologist communities in different geographic regions. It may be that many resident physicians stay within the geographic vicinity of their training programs and that these programs likely emphasize the importance of diagnosing LVI in their own way and that this training leads to the regional variations. Likewise, variation in diagnosis of LVI was seen by population type and type of facility. Academic institutions were significantly more likely to diagnose LVI than patients undergoing resection at Community Cancer Programs, Comprehensive Cancer Centers, and Integrated Network Cancer Programs. Likewise, LVI was significantly more likely to be diagnosed with surrounding populations greater than 1,000,000 compared with smaller populations. It is interesting to note that those facilities more likely to have diagnosed LVI in their tumor specimens were also more likely to have a better OS in MVA modeling. Surgically resected patients with lung cancer from academic facilities had a significantly better OS than those patients from Community Cancer Programs and Comprehensive Community Centers, and showed a trend for better survival compared with Integrated Cancer Centers. Likewise, there was a significantly better OS for patients treated in larger populated areas. Likewise, surgically resected patients with lung cancer from the Mid-Atlantic area had better OS than 5 of the other 8 surrounding communities and was similar at the other 3. We can speculate that better pathologic diagnosis in the facilities/areas/populations may be associated with better OS due to increased use of adjuvant therapy if the surgical specimens are LVI+, but our exploratory analysis did not demonstrate any higher use of adjuvant chemotherapy or radiation depending on LVI status (Table E3) at these institutions. We postulate that the increased occurrence of LVI at these institutions may be a surrogate for better overall care for these patients.

Despite this diagnostic variance, it is interesting to note that LVI still has a strongly negative effect on OS by MVA and PSM. The PSM also demonstrated that LVI had a strongly negative effect on OS on all tumor differentiations as well as adenocarcinomas and squamous cell carcinomas. It was interesting to note that LVI's detrimental effect on OS was significant for smaller tumors and lower stages, but lost significance, but trended higher for advanced pathologic stages and tumor sizes (stage 3 - 1.04 [0.93-1.16], T3 - 1.09 [0.93-1.27], N2 - 1.04 [0.93-1.18], tumor size >4 cm - 1.06 [0.96-1.16]). We can speculate that LVI may lose its prognostic impact with more advanced presentations, but it may also be that LVI is underdiagnosed at these advanced stages as noted in our previous analysis.^33^ Table E4 analyzes the differences between those with an LVI diagnosis (whether positive or negative) and those without an LVI diagnosis. LVI is more likely to be undiagnosed in tumors with positive/unknown margins, poor differentiation, larger tumor size, greater number of positive nodes, and higher T/N stages.

Although there are varying definitions for the adequacy of pathologic node staging and of complete resection^34^ for NSCLC, we chose the definition of those with examination of a minimum of 10 lymph nodes irrespective of node station location as per the American College of Surgeons Commission on Cancer NSCLC quality of surveillance criteria because this is the source of our data.^16^ We thought that it was important to assess patients with an adequate node dissection in our population (LB adequate node dissection) because it allowed us to investigate the impact of LVI without concerns of underdiagnosed lymph node involvement. Of note, the American College of Surgeons Commission on Cancer recently updated its recommendation for an adequate lymph node evaluation from at least 10 nodes examined to at least 1 hilar station and 3 mediastinal stations.^35^ These recommendations were based on the theoretical concept that lymph node location may be more beneficial than total number of nodes examined, but a recent retrospective review of 9749 patients noted that this updated definition offered minimal benefit compared with the past count-based approach.^34^ It should be noted that only 39.5% of patients undergoing LB had an adequate node dissection in our investigation. Likewise, even in a more recent prospective investigation accruing patients between 2014 and 2019, only 53% of patient enrolled in the ALCHEMIST study had an adequate node dissection despite the protocol requiring only a sampling of 1 N1 node (node stations 10 or 11) and any 3 mediastinal node stations regardless of primary tumor location.^36^ Positive surgical resection margins were noted in 4.0% of patients (2.9% and 7.4% in those who were LVI negative and positive, respectively). Interestingly, the rate of positive margins (4.0%) was similar to an early analysis using the NCDB from the years 2004 to 2011.^37^ It is hoped that lymph node resection kits^38^ will help standardize and improve lymph node examinations in the future. Our investigation demonstrates that any lesion undergoing LB adequate node dissection shown to have LVI may benefit from the consideration of systemic therapy, especially now that immunotherapy has shown a great impact on event-free survival/disease-free survival in recent studies.^14^, ^15^, ^16^, ^17^, ^18^ Although LVI was less likely to be seen in advanced stages and larger tumor sizes, these patients are already considered to be candidates for systemic therapy. However, we believe that our results in patients with smaller tumors less than 4 cm and even less than 2 cm should merit consideration of systemic therapy based on the presence of LVI as a solitary risk factor as noted in Figure 3, *D*.

### Study Limitations

**Figure E3:**
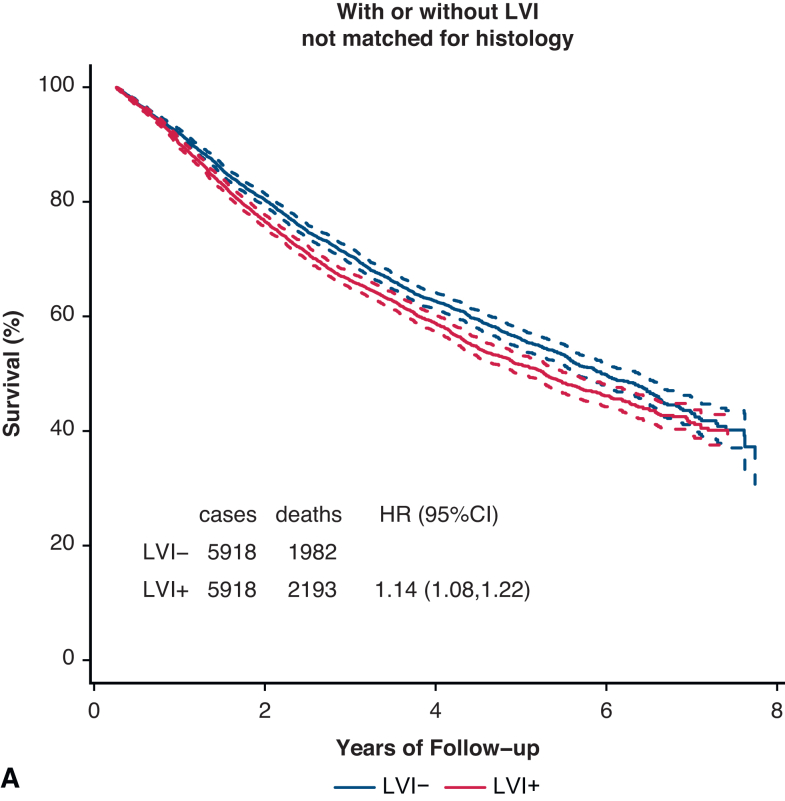

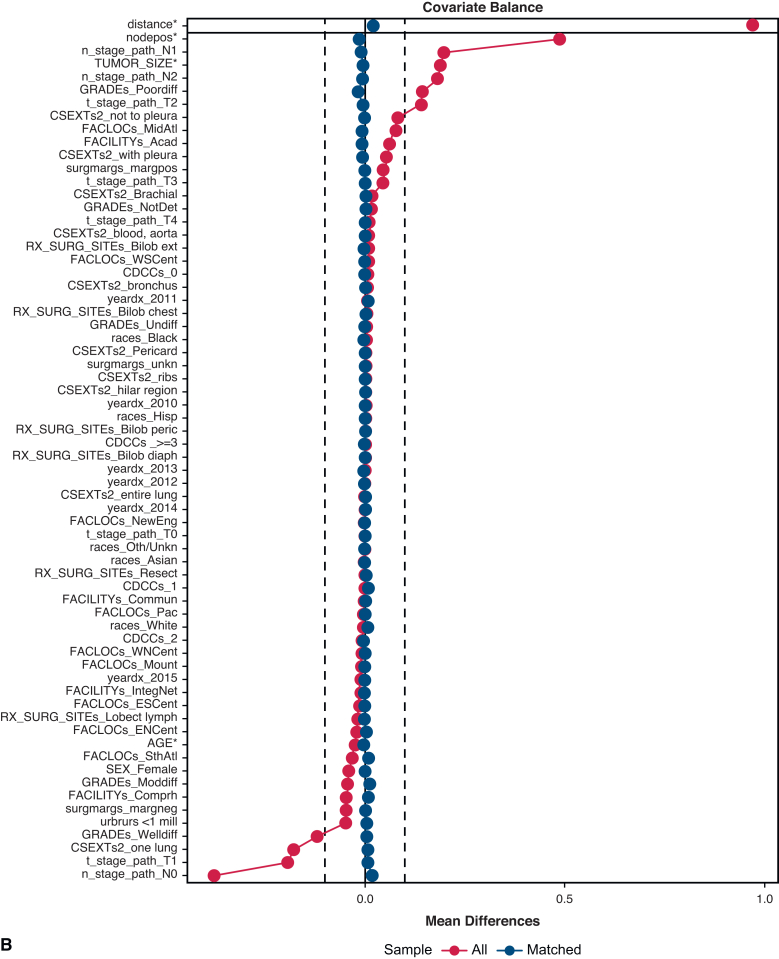
A, Propensity score analysis-adjusted overall survival of patients with and without LVI excluding histology. B, Love plot showing matching of characteristics used for propensity match for patients with and without LVI excluding histology. *LVI*, Lymphatic vascular invasion; *HR*, hazard ratio; *CI*, confidence interval.

There are limitations to this study, including nonspecific location of nodal examinations, lack of information pertaining to patient comorbidities, retrospective design, absence of recurrence patterns, and lack of explanation for modality choices. Additionally, there was no information concerning actionable mutations.^39^ Furthermore, although there was information concerning margin positivity, there was no information concerning completeness of resection by the IASLC criteria.^40^ The IASLC's definition of complete resection is quite strict and included the following: not only the absence of gross/microscopic disease but also at least 3 nodes removed from both mediastinal (sampling of subcarinal nodes mandatory)/hilar locations, no extracapsular nodal extension, negativity of the highest mediastinal node, and a negative pleural lavage cytology. Three large retrospective reviews of IASLC data,^41^ single institution,^42^ and multiple US institutions^43^ validated the IASLC definition with significantly and progressively lower OS in patients with complete, uncertain, and incomplete resection margins with the exception of complete and uncertain margins in node-negative patients in one of the investigations.^41^ By using this IASLC definition of complete resection and using intraoperative surgical margin assessment, it would be interesting to see if the impact of LVI would remain the same. To be complete, we decided to include all non–small histologies in our analysis. Large-cell neuroendocrine carcinoma was included in this analysis because it was still classified as a non–small cell lung cancer during the years of our study.^44^ Because the lung cancer histology may not be diagnosed on small biopsy specimens, we conducted a sensitivity analysis without histology to investigate whether LVI + would have the same effect on OS (Figure E3, *A* for OS and Figure E3, *B* for the associated Love plot). Excluding histology had no effect on our findings that LVI has a significantly detrimental effect on OS (HR, 1.15; 1.08-1.22) including histology and (HR, 1.14; 1.08-1.22) excluding histology. Although only 8.4% of our population did not have a diagnosis of LVI (whether positive or negative), this population was more likely to have advanced tumor/nodal stages, poor differentiation, and positive/unknown margins (Table E4). The lack of LVI diagnosis in these advanced presentations may have been a reason why LVI did not have a significant OS decrement in advanced T/N/overall stage and larger tumor sizes. However, despite these limitations and the lack of uniformity of LVI diagnosis in our patient population, LVI had an impact on OS for all surgical groups examined in this study. We think our study raises awareness of LVI as a single factor that may be used to assess patients for consideration of adjuvant therapy.

## Conclusions

LVI+ was associated with positive nodes, but the diagnosis of LVI+ differed based on hospital location/type and population. Despite the varying diagnosis, LVI was associated with a decrement in OS that was independent of histology and differentiation, and OS was noted to be significantly worse with LVI for tumor sizes less than 4 cm as well as pathologic stages T2, N0/N1, and stages 1 and 2. LVI is an important prognostic factor for patients with NSCLC and must be reported consistently, especially in lower-stage disease for which it may help consideration of adjuvant therapy. Once consistency is achieved, our study suggests that LVI should be considered as a factor for lung cancer staging. Better pathologic diagnosis of LVI may identify small (<4 cm) tumors that may benefit from adjuvant chemotherapy ± adjuvant immunotherapy.

## Conflict of Interest Statement

The authors reported no conflicts of interest.

The *Journal* policy requires editors and reviewers to disclose conflicts of interest and to decline handling or reviewing manuscripts for which they may have a conflict of interest. The editors and reviewers of this article have no conflicts of interest.

## Figures and Tables

**Table E1:** Factors associated with overall survival in our patient population of surgically resected patients with non−small cell lung cancer: ANOVA test

Variables	Likelihood ratio	*P* value
Age	463.67	<.001
Sex	161.18	<.001
Races	20.11	<.001
Charlson comorbidity (CDCC)	132.19	<.001
Histology	110.20	<.001
Tumor grade	58.49	<.001
Tumor size	46.05	<.001
Number nodes positive	87.16	<.001
Facility type	29.25	<.001
Facility location	48.83	<.001
Urban/rural population	9.40	.002
Year diagnosed	14.72	.012
pT stage	178.88	<.001
pN stage	371.19	<.001
Surgical margins	43.88	<.001
Type of surgery	10.95	.052
Extension	14.01	.081
Lung radiation	44.32	<.001
Chemotherapy	78.56	<.001
LVI	44.55	<.001

*CDCC*, Charlston-Deyo Score; *LVI*, lymphatic vascular invasion; *ANOVA*, analysis of variance.

**Table E2:** Variables with HRs (95% CIs) from the propensity matches for LVI

Variable	HR (95% CI) for OS
Tumor size, cm	
≤2	1.20 (1.03-1.40)
2.1-4	1.16 (1.06-1.27)
>4	1.06 (0.96-1.16)
Stage	
Stage 1	1.35 (1.19-1.53)
Stage 2	1.15 (1.04-1.27)
Stage 3	1.04 (0.93-1.16)
T stage	
T1	1.18 (1.05-1.32)
T2	1.15 (1.06-1.24)
T3	1.09 (0.93-1.27)
N stage	
N0	1.28 (1.16-1.41)
N1	1.15 (1.03-1.27)
N2	1.04 (0.93-1.18)
Histology	
Adenocarcinoma	1.18 (1.09-1.29)
Squamous cell carcinoma	1.14 (1.03-1.27)
Differentiation	
Well	1.25 (0.91-1.71)
Moderate	1.14 (1.04-1.25)
Poor	1.13 (1.04-1.22)

HRs represent impact of LVI on OS. *HR*, Hazard ratio; *CI*, confidence interval; *OS*, overall survival; *LVI*, lymphatic vascular invasion.

**Table E3:** Use of radiation and/or chemotherapy by LVI status

Variable	Patients, n	LVIneg	LVIpos	OR (95% CI)	*P* value
Facility: academic	11,894	8782	3112		
Chemotherapy: yes	3934	2285	1649	3.20 (2.94-3.49)	<.001
Chemotherapy: no	7960	6497	1463		
Radiation therapy: yes	1175	632	543	2.73 (2.41-3.08)	<.001
Radiation therapy: no	10719	8150	2569		
Facility: community cancer program	1612	1250	362		
Chemotherapy: yes	630	410	220	3.17 (2.50-4.05)	<.001
Chemotherapy: no	982	840	142		
Radiation therapy: yes	234	135	99	3.11 (2.32-4.16)	<.001
Radiation therapy: no	1378	1115	263		
Facility: comprehensive community center	10,978	8644	2334		
Chemotherapy: yes	3647	2307	1340	3.70 (3.37-4.07)	<.001
Chemotherapy: no	7331	6337	994		
Radiation therapy: yes	1127	662	465	3.00 (2.64-3.41)	<.001
Radiation therapy: no	9851	7982	1869		
Facility: integrated network cancer program	4358	3390	969		
Chemotherapy: yes	1497	949	548	3.35 (2.89-3.88)	<.001
Chemotherapy: no	2861	2440	421		
Radiation therapy: yes	480	283	197	2.80 (2.30-3.41)	<.001
Radiation therapy: no	3878	3106	772		
Facility location: Mid-Atlantic	5308	3663	1645		
Chemotherapy: yes	1856	966	890	3.29 (2.91-3.72)	<.001
Chemotherapy: no	3452	2697	755		
Radiation therapy: yes	561	281	280	2.47 (2.07-2.95)	<.001
Radiation therapy: no	4747	3382	1365		
Facility location: East North Central	5087	3995	1092		
Chemotherapy: yes	1818	1160	658	3.71 (3.23-4.26)	<.001
Chemotherapy: no	3269	2835	434		
Radiation therapy: yes	588	356	232	2.76 (2.30-3.30)	<.001
Radiation therapy: no	4499	3639	860		
Facility location : East South Central	2447	1937	510		
Chemotherapy: yes	778	504	274	3.30 (2.70-4.04)	<.001
Chemotherapy: no	1669	1433	236		
Radiation therapy: yes	229	130	99	3.35 (2.52-4.44)	<.001
Radiation therapy: no	2218	1807	411		
Facility location: Mountain	985	797	188		
Chemotherapy: yes	279	195	84	2.49 (1.79-3.47)	<.001
Chemotherapy: no	706	602	104		
Radiation therapy: yes	79	45	34	3.69 (2.28-5.94)	<.001
Radiation therapy: no	906	752	154		
Facility location: New England	1592	1220	372		
Chemotherapy: yes	555	342	213	3.44 (2.71-4.38)	<.001
Chemotherapy: no	1037	878	159		
Radiation therapy: yes	184	104	80	2.94 (2.13-4.04)	<.001
Radiation therapy: no	1408	1116	292		
Facility location: Pacific	2786	2155	631		
Chemotherapy: yes	859	521	338	3.62 (3.01-4.36)	<.001
Chemotherapy: no	1927	1634	293		
Radiation therapy: yes	274	158	116	2.85 (2.20-3.68)	<.001
Radiation therapy: no	2512	1997	515		
Facility location: South Atlantic	6670	5269	1401		
Chemotherapy: yes	2205	1412	793	3.56 (3.15-4.03)	<.001
Chemotherapy: no	4465	3857	608		
Radiation therapy: yes	686	403	283	3.06 (2.59-3.60)	<.001
Radiation therapy: no	5984	4866	1118		
Facility location: West North Central	2137	1677	460		
Chemotherapy: yes	731	493	238	2.57 (2.09-3.18)	<.001
Chemotherapy: no	1406	1184	222		
Radiation therapy: yes	234	146	88	2.48 (1.86-3.30)	<.001
Radiation therapy: no	1903	1531	372		
Facility location: West South Central	1830	1352	478		
Chemotherapy: yes	627	358	269	3.57 (2.88-4.45)	<.001
Chemotherapy: no	1203	994	209		
Radiation therapy: yes	181	89	92	3.38 (2.47-4.63)	<.001
Radiation therapy: no	1649	1263	386		
Urban/rural >1 million local population (ref)	15,111	11,312	3799		
Chemotherapy: yes	5034	2975	2059	3.32 (3.07-3.60)	<.001
Chemotherapy: no	10,077	8337	1740		
Radiation therapy: yes	1597	881	716	2.75 (2.47-3.06)	<.001
Radiation therapy: no	13,514	10,431	3083		
Urban/rural <1 million local population	13,731	10,753	2978		
Chemotherapy: yes	4674	2976	1698	3.47 (3.19-3.77)	<.001
Chemotherapy: no	9057	7777	1280		
Radiation therapy: yes	1419	831	588	2.94 (2.62-3.29)	<.001
Radiation therapy: no	12,312	9922	2390		

*LVI*, Lymphatic vascular invasion; *OR*, odds ratio; *CI*, confidence interval.

**Table E4:** Differences between populations with a diagnosis of LVI (whether positive or negative) and those with a missing LVI diagnosis

Comparison of patients with no diagnosis (not available) vs those with diagnosis (+ or −)	Undiagnosed	Diagnosed		Overall
	(1)	(2)	*P* value	
	(n = 2653)	(n = 28,842)	(1) vs (2)	(N = 31,495)
Age at diagnosis, y			**.035**	
Mean (SD)	66.4 (9.40)	66.8 (9.34)		66.8 (9.35)
Median [min-max]	67.0 [40.0-90.0]	67.0 [40.0-90.0]		67.0 [40.0-90.0]
Sex			.61	
Male	1286 (48.5%)	13,827 (47.9%)		15,113 (48.0%)
Female	1367 (51.5%)	15,015 (52.1%)		16,382 (52.0%)
Race/ethnicity			**.028**	
White	2134 (80.4%)	23,814 (82.6%)		25,948 (82.4%)
Hispanic	84 (3.2%)	683 (2.4%)		767 (2.4%)
Black	231 (8.7%)	2286 (7.9%)		2517 (8.0%)
Asian	80 (3.0%)	858 (3.0%)		938 (3.0%)
Other	124 (4.7%)	1201 (4.2%)		1325 (4.2%)
CDCC			**<.001**	
0	1480 (55.8%)	14,687 (50.9%)		16,167 (51.3%)
1	822 (31.0%)	9750 (33.8%)		10,572 (33.6%)
2	270 (10.2%)	3314 (11.5%)		3584 (11.4%)
≥3	81 (3.1%)	1091 (3.8%)		1172 (3.7%)
Histology			**.008**	
Adenocarcinoma	1492 (56.2%)	16,549 (57.4%)		18,041 (57.3%)
Adenosquamous	64 (2.4%)	720 (2.5%)		784 (2.5%)
Bronchoalveolar	128 (4.8%)	1569 (5.4%)		1697 (5.4%)
Large cell	102 (3.8%)	758 (2.6%)		860 (2.7%)
Large cell neuroendocrine	38 (1.4%)	359 (1.2%)		397 (1.3%)
Pulmonary sarcomatoid	25 (0.9%)	255 (0.9%)		280 (0.9%)
Salivary gland	9 (0.3%)	49 (0.2%)		58 (0.2%)
Squamous	795 (30.0%)	8583 (29.8%)		9378 (29.8%)
Grade			**<.001**	
Well differentiated	220 (8.3%)	3704 (12.8%)		3924 (12.5%)
Moderately differentiated	1059 (39.9%)	12,935 (44.8%)		13,994 (44.4%)
Poor differentiated	1012 (38.1%)	10,253 (35.5%)		11,265 (35.8%)
Undifferentiated	52 (2.0%)	345 (1.2%)		397 (1.3%)
Not determined	310 (11.7%)	1605 (5.6%)		1915 (6.1%)
Tumor size, mm			**<.001**	
Mean (SD)	37.9 (27.2)	34.6 (27.9)		34.8 (27.9)
Median [min-max]	32.0 [1.00-800]	28.0 [1.00-950]		29.0 [1.00-950]
Nodes positive			**<.001**	
Mean (SD)	1.15 (2.67)	0.876 (2.25)		0.899 (2.29)
Median [min-max]	0 [0-45.0]	0 [0-61.0]		0 [0-61.0]
Facility type			**<.001**	
Academic	1165 (43.9%)	11,894 (41.2%)		13,059 (41.5%)
Community	188 (7.1%)	1612 (5.6%)		1800 (5.7%)
Comprehensive	977 (36.8%)	10,978 (38.1%)		11,955 (38.0%)
Integrated network cancer program	323 (12.2%)	4358 (15.1%)		4681 (14.9%)
Facility location			**<.001**	
Mid-Atlantic	464 (17.5%)	5308 (18.4%)		5772 (18.3%)
East North Central	522 (19.7%)	5087 (17.6%)		5609 (17.8%)
East South Central	173 (6.5%)	2447 (8.5%)		2620 (8.3%)
Mountain	58 (2.2%)	985 (3.4%)		1043 (3.3%)
New England	124 (4.7%)	1592 (5.5%)		1716 (5.4%)
Pacific	347 (13.1%)	2786 (9.7%)		3133 (9.9%)
South Atlantic	614 (23.1%)	6670 (23.1%)		7284 (23.1%)
West North Central	205 (7.7%)	2137 (7.4%)		2342 (7.4%)
West South Central	146 (5.5%)	1830 (6.3%)		1976 (6.3%)
Urban/rural			.56	
>1 million	1406 (53.0%)	15,111 (52.4%)		16,517 (52.4%)
<1 million	1247 (47.0%)	13,731 (47.6%)		14,978 (47.6%)
Year of diagnosis			**<.001**	
2010	451 (17.0%)	3781 (13.1%)		4232 (13.4%)
2011	444 (16.7%)	4106 (14.2%)		4550 (14.4%)
2012	448 (16.9%)	4324 (15.0%)		4772 (15.2%)
2013	448 (16.9%)	4720 (16.4%)		5168 (16.4%)
2014	418 (15.8%)	5383 (18.7%)		5801 (18.4%)
2015	444 (16.7%)	6528 (22.6%)		6972 (22.1%)
t-stage pathology			**<.001**	
T0	12 (0.5%)	18 (0.1%)		30 (0.1%)
T1	1052 (39.7%)	12,912 (44.8%)		13,964 (44.3%)
T2	1241 (46.8%)	12,882 (44.7%)		14,123 (44.8%)
T3	317 (11.9%)	2820 (9.8%)		3137 (10.0%)
T4	31 (1.2%)	210 (0.7%)		241 (0.8%)
n-stage pathology			**<.001**	
N0	1708 (64.4%)	21,112 (73.2%)		22,820 (72.5%)
N1	573 (21.6%)	4710 (16.3%)		5283 (16.8%)
N2	372 (14.0%)	3020 (10.5%)		3392 (10.8%)
Surgical margins			**<.001**	
Negative	2457 (92.6%)	27,565 (95.6%)		30,022 (95.3%)
Positive	144 (5.4%)	1146 (4.0%)		1290 (4.1%)
Unknown	52 (2.0%)	131 (0.5%)		183 (0.6%)
Site of surgery			**<.001**	
Lobectomy with lymph node	2198 (82.8%)	24,991 (86.6%)		27,189 (86.3%)
Bilobectomy extended	86 (3.2%)	798 (2.8%)		884 (2.8%)
Bilobectomy with chest wall	65 (2.5%)	474 (1.6%)		539 (1.7%)
Bilobectomy with diaphragm	3 (0.1%)	26 (0.1%)		29 (0.1%)
Bilobectomy with pericardium	7 (0.3%)	52 (0.2%)		59 (0.2%)
Resection	294 (11.1%)	2501 (8.7%)		2795 (8.9%)
Collaborative stage extension			**<.001**	
One lung	1825 (68.8%)	20,888 (72.4%)		22,713 (72.1%)
Bronchus	69 (2.6%)	535 (1.9%)		604 (1.9%)
Entire lung	1 (0.0%)	29 (0.1%)		30 (0.1%)
Hilar region	45 (1.7%)	294 (1.0%)		339 (1.1%)
Blood-aorta	28 (1.1%)	223 (0.8%)		251 (0.8%)
Pleura	514 (19.4%)	5646 (19.6%)		6160 (19.6%)
Ribs	30 (1.1%)	123 (0.4%)		153 (0.5%)
Brachial	132 (5.0%)	1043 (3.6%)		1175 (3.7%)
Pericardial	9 (0.3%)	61 (0.2%)		70 (0.2%)
Any lung radiation			**<.001**	
No	2149 (81.0%)	25,826 (89.5%)		27,975 (88.8%)
Yes	504 (19.0%)	3016 (10.5%)		3520 (11.2%)
Any lung chemotherapy			**<.001**	
No	1452 (54.7%)	19,134 (66.3%)		20,586 (65.4%)
Yes	1201 (45.3%)	9708 (33.7%)		10,909 (34.6%)

*t*-tests were used for continuous variables and the Fisher test for categorical variables. *P* values comparing those with no LVI diagnosis vs those with an LVI diagnosis. *LVI*, Lymphatic vascular invasion; *CDCC*, Charlston-Deyo Score; *SD*, standard deviation.
